# Novel functional hepatitis C virus glycoprotein isolates identified using an optimized viral pseudotype entry assay

**DOI:** 10.1099/jgv.0.000537

**Published:** 2016-09

**Authors:** Richard A. Urbanowicz, C. Patrick McClure, Barnabas King, Christopher P. Mason, Jonathan K. Ball, Alexander W. Tarr

**Affiliations:** ^1^​ School of Life Sciences, The University of Nottingham, Nottingham University Hospitals NHS Trust, Nottingham, UK; ^2^​ NIHR Nottingham Digestive Diseases Biomedical Research Unit, The University of Nottingham, Nottingham University Hospitals NHS Trust, Nottingham, UK

**Keywords:** Hepatitis C virus, Ebola virus, Pseudoparticle, Pseudotype, Pseudovirus, envelope glycoproteins, HCVpp

## Abstract

Retrovirus pseudotypes are a highly tractable model used to study the entry pathways of enveloped viruses. This model has been extensively applied to the study of the hepatitis C virus (HCV) entry pathway, preclinical screening of antiviral antibodies and for assessing the phenotype of patient-derived viruses using HCV pseudoparticles (HCVpp) possessing the HCV E1 and E2 glycoproteins. However, not all patient-isolated clones produce particles that are infectious in this model. This study investigated factors that might limit phenotyping of patient-isolated HCV glycoproteins. Genetically related HCV glycoproteins from quasispecies in individual patients were discovered to behave very differently in this entry model. Empirical optimization of the ratio of packaging construct and glycoprotein-encoding plasmid was required for successful HCVpp genesis for different clones. The selection of retroviral packaging construct also influenced the function of HCV pseudoparticles. Some glycoprotein constructs tolerated a wide range of assay parameters, while others were much more sensitive to alterations. Furthermore, glycoproteins previously characterized as unable to mediate entry were found to be functional. These findings were validated using chimeric cell-cultured HCV bearing these glycoproteins. Using the same empirical approach we demonstrated that generation of infectious ebolavirus pseudoviruses (EBOVpv) was also sensitive to the amount and ratio of plasmids used, and that protocols for optimal production of these pseudoviruses are dependent on the exact virus glycoprotein construct. These findings demonstrate that it is crucial for studies utilizing pseudoviruses to conduct empirical optimization of pseudotype production for each specific glycoprotein sequence to achieve optimal titres and facilitate accurate phenotyping.

## Introduction

Chronic hepatitis C virus (HCV) infection affects approximately 3 % of the world’s population. Infection can result in fibrosis, decompensated liver disease and hepatocellular carcinoma. Genetic variability is a key feature of HCV, with viral isolates classified into six major genotypes that differ by up to 30 % at the nucleotide level. The genomes of isolates within a genotype can differ by up to 15 %. Furthermore the virus exists as a quasispecies population in an infected host, with approximately 1 % of the genome varying between strains (Nasu *et al.*, 2011). This extreme variability contributes to the persistent nature of HCV infection and presents a significant barrier to developing broadly active therapies and vaccines.

HCV possesses a complex entry pathway mediated by the HCV E1 and E2 glycoproteins, which bind to receptors and facilitate membrane fusion [reviewed in Tarr *et al*. (2015)]. These glycoproteins display the greatest level of genetic diversity in the HCV genome (Nasu *et al.*, 2011), yet receptor interactions are highly conserved between genetically distinct strains (Gottwein *et al.*, 2009; Lavillette *et al.*, 2005). This conserved entry pathway highlights the potential for vaccine-induced broadly neutralizing antibodies. Development of an effective vaccine remains a key challenge for the eradication of HCV infection and assessing the potency of neutralizing antibodies against strains representing *in vivo* infections is critical to this goal. *In vitro* assessment of the breadth of HCV neutralization has predominantly utilized two assays. First, the cell-culture replicating molecular clone JFH-1 and chimeras derived from this virus possessing the structural genes of other viruses provide a method of assessing neutralization of small panels of genetically diverse viruses (Carlsen *et al.*, 2014; Pedersen *et al.*, 2013). Second, retroviral pseudotypes (HCV pseudoparticles; HCVpp), which can be rapidly reconstituted with the HCV E1 and E2 glycoproteins of diverse strains, permit assessment of neutralization against a wide range of virus isolates (Bailey *et al.*, 2015; Fafi-Kremer *et al.*, 2010; Osburn *et al.*, 2014; Urbanowicz *et al.*, 2015). This assay was instrumental in identifying common HCV entry pathways utilizing conserved host cellular receptors that interact with the HCV glycoproteins (Bartosch *et al.*, 2003c; Evans *et al.*, 2007; Lavillette *et al.*, 2005).

Retroviral pseudoparticles have been utilized to investigate the nature of infectious isolates representing the quasispecies variants in natural HCV infections (Fafi-Kremer *et al.*, 2010; Osburn *et al.*, 2014). However, variable success recovering functioning glycoprotein sequences from HCV-infected individuals between studies indicated that *in vitro* artefacts might contribute to the failure to identify infectious clones in some patients. This could lead to bias in the phenotype of HCV strains that are selected for analysis of entry inhibitors and therefore provide an unrepresentative view of the properties of glycoproteins representing circulating HCV strains. While the methods for producing HCVpp are generally standardized (Bartosch *et al.*, 2003a; Hsu *et al.*, 2003; Tarr *et al.*, 2007), successful generation of infectious pseudotypes is dependent on many factors [reviewed in King *et al*. (2016)].

We previously reported that about 24–27 % of glycoprotein clones isolated from chronic and acute infections yield infectious HCVpp (Lavillette *et al.*, 2005; Urbanowicz *et al.*, 2015). It was observed that even closely related sequences contrasted in their ability to produce infectious particles. To investigate this further, the properties of HCV pseudoparticles bearing patient-derived HCV glycoproteins were investigated. It was discovered that the infectivity of patient-derived E1/E2 glycoproteins previously determined to be non-functional is influenced by expression protocols used to generate HCVpp. Furthermore, infectivity was dependent on the species of the packaging construct used. To determine if these potential artefacts occur in pseudotypes representing other variable RNA viruses, we also compared infectivity of pseudoviruses incorporating genetically related ebolavirus glycoproteins (EBOVpv). Varying the EBOVpv genesis protocol had similar effects on EBOVpv on infectivity. This work has implications for future studies of not only HCV patient strains, but also for the investigation of other viruses that rely on pseudotyped entry models.

## Results

### Infectivity phenotype of HCV E1/E2 pseudoparticles is dependent on a fine balance of plasmid expression

**Fig. 1.**
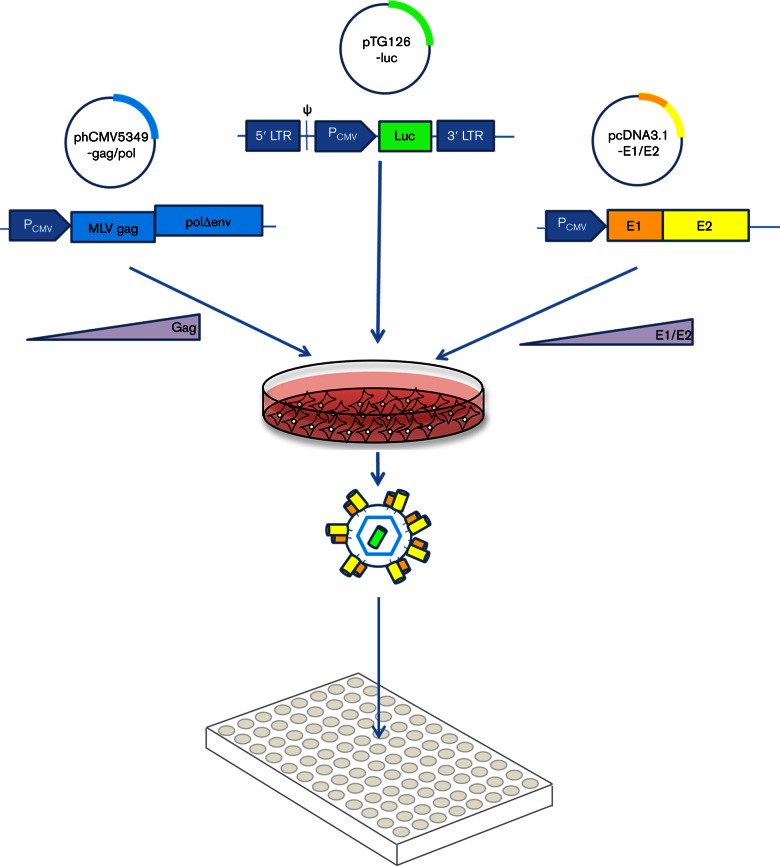
Experimental setup for HCV pseudotype production in this study. Transfections were prepared with a fixed amount of the reporter plasmid pTG126, encoding an RNA transcript with a packaging signal (ψ) and a luciferase reporter gene under the control of a CMV IE promoter, with flanking MLV long terminal repeats (LTRs). This plasmid was co-transfected with varying quantities of both the E1/E2-encoding plasmid (derived from pcDNA3.1) and the MLV Gag/Pol-encoding packaging plasmid (phCMV5349) to create a matrix of transfections for each patient-derived clone. HEK293T cells were transfected with the mixture of plasmids, and the pseudotypes generated were used to infect HuH7 cells in a 96-well format. Infectivity was determined as RLU from the expressed reporter gene in the HuH7 cells.

**Fig. 2.**
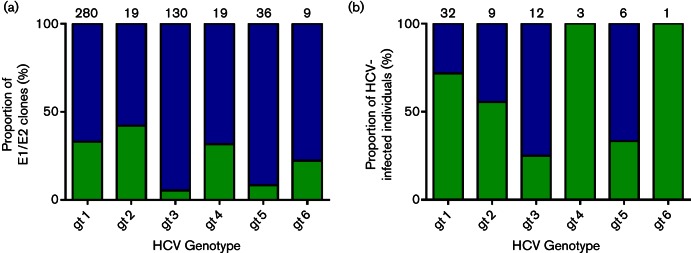
Frequency of recovery of functional HCV glycoproteins using an MLV-based retroviral pseudoparticle assay. Patient isolates were screened using standard assay parameters as previously described (Tarr *et al.*, 2007). (a) From a total of 63 patients, 118 E1/E2 clones were defined as functional in this assay (Urbanowicz *et al.*, 2015). The majority of E1/E2 clones (76 %) were non-functional in this assay. The total number of clones screened for each genotype is noted above each bar. The proportion of functional clones (green) and the proportion of non-functional clones (blue) is displayed. When segregated by genotype, the proportion of functional genotype 3 and genotype 5 clones was lower than genotypes 1, 2 or 6. (b) Frequency of recovery of functional HCV glycoproteins from HCV-infected individuals. The total number of individuals infected with each genotype is highlighted above each bar. Again, the proportion of patients with functional clones (green) was lower for genotype 3 and genotype 5.

HCV pseudoparticles were generated using a standard three-plasmid system, utilizing murine leukaemia virus backbone/luciferase reporter plasmids (Bartosch *et al.*, 2003a) and HCV E1/E2 proteins expressed in *cis* in a pcDNA3.1 plasmid (Tarr *et al.*, 2007) (Fig. 1). A panel of 493 E1/E2 clones from 63 different HCV-infected patients were screened for infectivity, as previously described (Urbanowicz *et al.*, 2015). Of these, only 118 clones (24 %) were identified as being infectious in our entry assay (Fig. 2). Infectious clones were not absolutely segregated by genotype or subtype, suggesting that lack of infectivity is dependent on isolate-specific mutations in these genes. However, in some cases all E1/E2 clones recovered from individual patients were non-infectious. The proportion of infectious clones from patients harbouring genotype 3 (5 %) and genotype 5 (8 %) virus populations were lower than other genotypes. Repeated assays with these clones determined the batch-to-batch variation in luciferase signal to be <20 % for an individual glycoprotein clone.

**Fig. 3.**
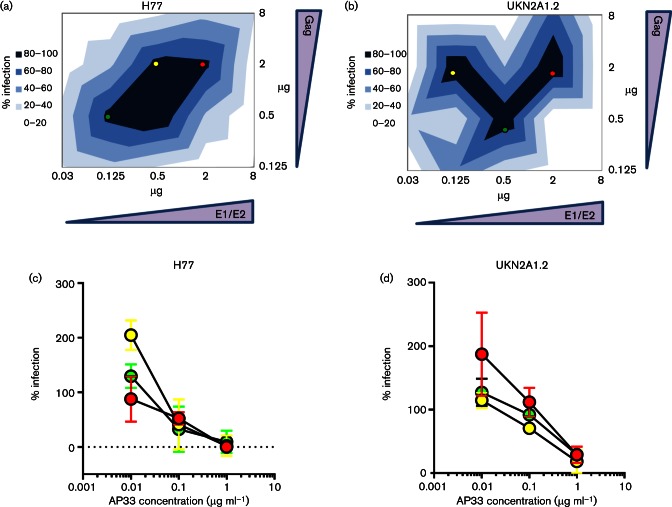
Plasmid quantity affects the infectivity of pseudoparticles generated with standard reference HCV envelope glycoproteins. (a and b) Transfection matrices were prepared for H77 (Owsianka *et al.*, 2006) and clone UKN2A1.2 (Lavillette *et al.*, 2005) with the resulting pseudoparticle preparations used to infect HuH7 cells. Graphs are presented as x-y contour plots of plasmid concentrations, with increasing normalized luciferase signal indicated by darkening blue colour. Signals were normalized against the maximal signal achieved for either H77 (~10 000 RLU), UKN2A1.2 (~5000 RLU) or that achieved with the negative control preparation (ΔE1/E2; ~100 RLU). Shades of blue represent the normalized infectivity data stratified into 20 % increments. HCVpp preparations with high signal were selected for neutralization assays (red, yellow and green points). (c and d) Neutralization of HCVpp prepared with different plasmid concentrations. Preparations were incubated with defined concentrations of the HCV-neutralizing mouse monoclonal antibody AP33. No differences were observed in the neutralization sensitivity of the particles.

Having observed this discrepancy in infectivity between genetically similar clones we investigated whether the amount of HCV glycoprotein construct delivered to the transfected cells influenced infectivity of the particles produced. A series of transfections was performed with a matrix of serial dilutions of the plasmid preparations of both the H77c (AF011751) reference consensus sequence and the patient-derived clone UKN2A1.2 (Fig. 3). Using supernatants from cells transfected with a fixed amount of the luciferase reporter gene and varying amounts of the MLV packaging construct and the HCV E1/E2 glycoprotein construct, infectivity values were determined for H77c (Fig. 3a) and UKN2A1.2 (Fig. 3b). The ratio of plasmids encoding the packaging construct and the glycoprotein construct was critical to the observed infectivity – at the extremes of concentrations analysed, little luciferase signal was observed for either clone. Maximum infectivity for H77 HCVpp occurred when either 2 ug or 0.5 ug of each plasmid was delivered to HEK293T cells. While infectivity of H77c HCVpp tolerated a broad range of concentrations of E1/E2 and packaging plasmids, the combinations of plasmid concentrations that resulted in maximal HCVpp infectivity for UKN2A1.2 were more restricted (Fig. 3b). As it was plausible that the differences in plasmid quantities used for transfection affected the incorporation of glycoproteins into pseudoparticles, neutralization assays were performed on preparations of virus expressed with different amounts of plasmid (Fig. 3c, d). However, no significant differences in neutralization curves were observed.

### Differences in infectivity between closely related viruses using the HCVpp model

**Fig. 4.**
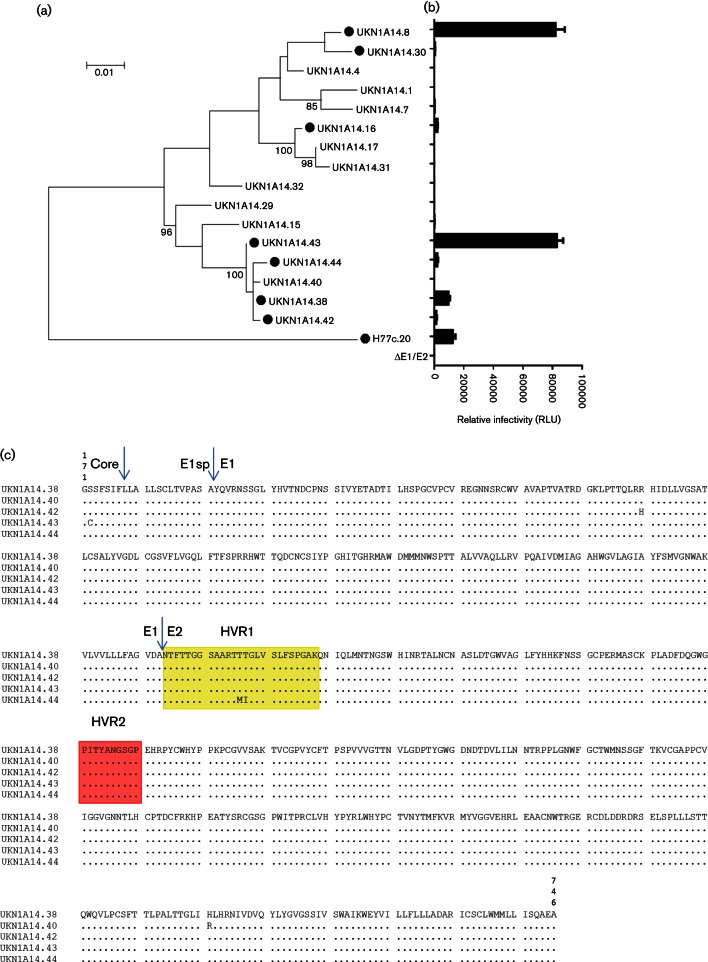
HCVpp infection mediated by E1/E2 genes of closely related members of an HCV quasispecies. (a) The E1/E2 coding region was amplified by RT-PCR from a genotype 1a-infected patient (equivalent to amino acids 171–746 of the reference H77c clone). Maximum likelihood phylogenetic analysis was performed using a WAG model as implemented in MEGA 6.0. Bootstrap analysis of phylogeny using 1000 replicates was performed and values >80 are highlighted. (b) MLV-based HCVpp each possessing an E1/E2 clone were screened for infectivity in HuH7 cells, using standard plasmid concentrations for HCVpp genesis. Seven of the 16 isolated clones (●) were defined as functional (greater than 3× background signal generated with pseudotypes in the absence of glycoprotein, labelled as ΔE1/E2), although relative infectivity values varied widely. (c) Alignment of the E1/E2 coding regions of patient-derived clones UKN1A14.38, UKN1A14.40, UKN1A14.42, UKN1A14.43 and UKN1A14.44. The hypervariable region 1 (HVR1) is highlighted in yellow, the HVR2 is highlighted in red. Amino acid polymorphisms are highlighted between the closely related strains. The protease cleavage sites between Core, E1 signal peptide, E1 and E2 regions are highlighted with arrows.

**Fig. 5.**
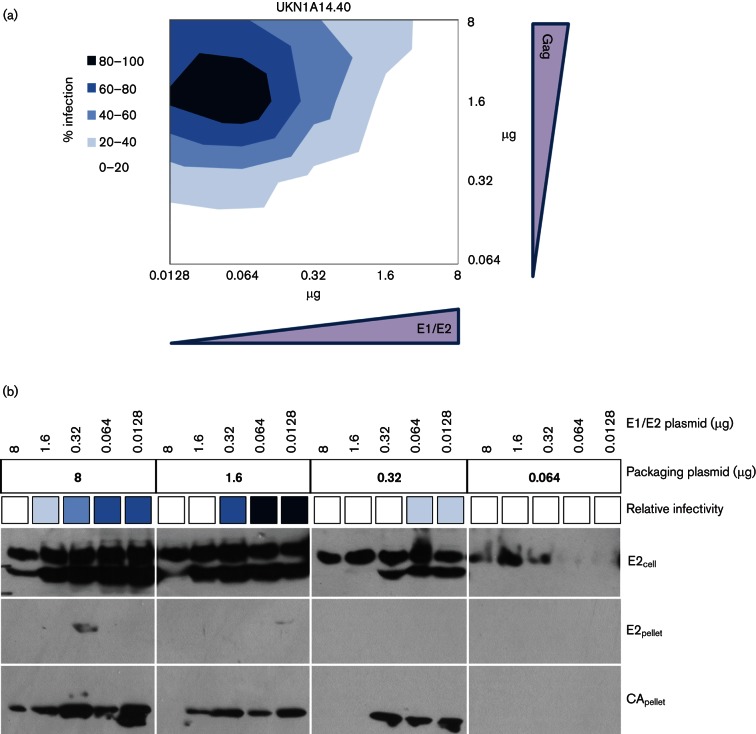

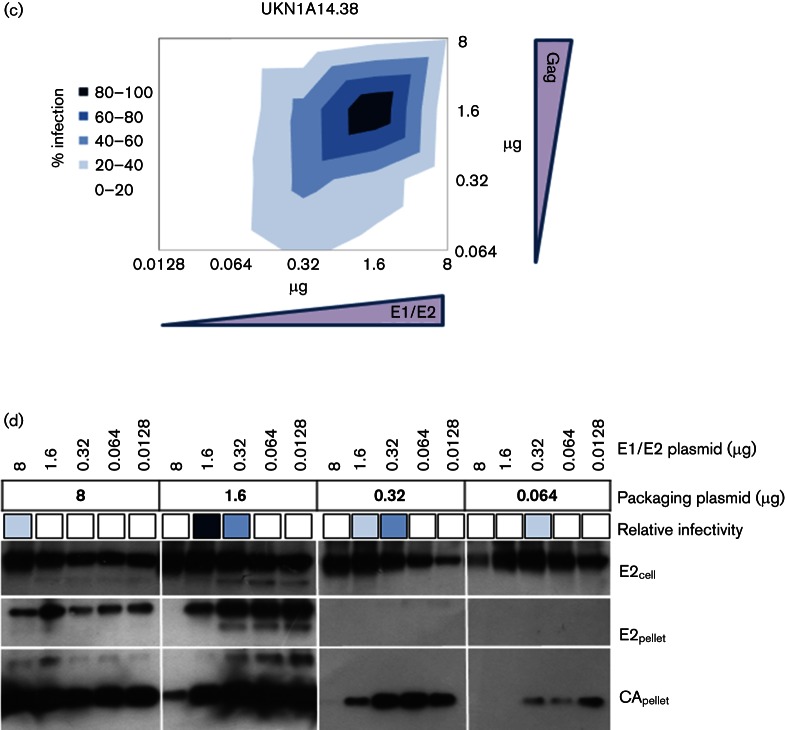
HCVpp infectivity and protein incorporation into pseudoparticles. (a) Infectivity of patient clone UKN1A14.40. Pseudoparticles were made with a matrix of varying quantities of the plasmids encoding packaging vector and E1/E2 glycoproteins. Relative infectivity was normalized to the greatest signal observed (~10 000 RLU) and the signal achieved with a negative control preparation (ΔE1/E2). The graph is presented as an x-y contour plot of Gag- and E1/E2-encoding plasmid concentrations. Greater infectivity is presented as darker blue, stratified into 20 % increments, normalized to maximal infectivity. (b) Western blotting of transfected cell lysates of cells producing UKN1A14.40 HCVpp, using anti-E2 mAbs AP33 and ALP98 (upper panel), and pelleted pseudoparticles with AP33/ALP98 (middle panel) or anti-MLV CA (lower panel). The relative infectivity for each combination of plasmids is displayed as the shade of blue corresponding to the scale presented in panel (a). Very little incorporation of E2 was observed, while capsids were detected when using greater concentrations of the packaging vector. (c) HCVpp infectivity pseudoparticles representing patient clone UKN1A14.38. Pseudoparticles were made with a matrix of varying quantities of the plasmids encoding packaging vector and E1/E2 glycoproteins. Relative infectivity was normalized to the greatest signal observed (~10 000 RLU). Shades of blue represent the normalized infectivity data in 20 % increments. (d) Western blotting of transfected cell lysates producing UKN1A14.38 HCVpp, using anti-E2 antibodies AP33 and ALP98 (upper panel), and pelleted pseudoparticles with AP33/ALP98 (middle panel) or anti-MLV CA (lower panel). The relative infectivity for each combination of plasmids is displayed as the shade of blue corresponding to the scale presented in panel (c). Incorporation of E2 was observed when either 8 or 1.6 µg of packaging vector was used, while capsids were detected at all concentrations of the packaging vector. Greater concentrations of glycoprotein plasmid resulted in reduced expression of capsid.

To determine if this experimental artefact contributed to the lack of infectivity observed for some patient isolates in our original panel, closely related sequences with different infectivity phenotypes in the HCVpp assay were analysed. Sixteen E1/E2 ORF clones from a well characterized treatment-naïve patient were selected [patient UKN1A14 (Brown *et al.*, 2005; Curran *et al.*, 2002) (Fig. 4a)]. HCVpp assays revealed contrasting patterns of infectivity with the glycoproteins using standard techniques (Fig. 4b). Nucleotide diversity across all the E1/E2 clones was <4 %. Clones that resulted in infectious particles could differ by as little as a single amino acid from those that did not. To determine if plasmid concentrations affected infectivity, two clones were selected: one that gave relative luminescence signal below the threshold of infectivity (UKN1A14.40), and one clone that gave a similar signal to the reference H77c (UKN1A14.38). These two clones differed by a single amino acid at position (H691R) in the E1/E2 coding region (Fig. 4c). Pseudoparticles possessing these glycoproteins were produced with varying amounts of E1/E2 plasmid and packaging vector. Despite the common plasmid backbone and promoter sequences in these clones, UKN1A14.40 (Fig. 5a) had contrasting patterns of infection to UKN1A14.38 (Fig. 5c). For UKN1A14.38, the greatest signal was achieved when using greater amounts (1.6 µg) of both packaging plasmid and glycoprotein-encoding plasmid, similar to those used for H77c. In contrast, the highest signal for UKN1A14.40 was achieved with 25-fold less E1/E2-encoding plasmid (0.064 µg) and 1.6 µg of packaging vector. Consistent with our earlier observation, UKN1A14.40 was not infectious when using standard assay parameters. To determine if infectivity was associated with better incorporation of MLV capsid or HCV glycoproteins into HCVpp, Western blots of sucrose cushion-purified pseudoparticles were performed and compared with the protein expression in producer 293T cell lysates (Fig. 5b, d). As expected, E2 was only observed in HCVpp preparations that contained detectable capsid protein. However, E2 was not detected in all preparations where capsid was detected, and detection of E2 did not correlate with the production of infectious pseudoparticles. For UKN1A14.40, E2 was only detectable when the greatest amount of MLV packaging plasmid was used, despite this coinciding with only 50–60 % maximal signal for this isolate. Similarly, infectivity was not always associated with detectable MLV-capsid protein in the pelleted pseudoparticles. It was observed that increasing the amount of the E1/E2-encoding plasmid resulted in reduced expression of capsid protein in producer cells. Glycoprotein expression in the UKN1A14.38-transfected cells was more readily detected. Comparison of the sequences of these clones revealed all glycoproteins possessed identical epitopes for mAbs AP33/ALP98 (aa412-420 and aa644-641, respectively) used for detection in Western blot (Fig. 4c). As such, detectable expression of UKN1A14.38 E1/E2 occurred over a larger range of plasmid concentrations, although the expression did not correlate directly with production of infectious HCVpp. The expression of E2 in Western blots of lysates of cells transfected with UKN1A14.40 or UKN1A14.38 was detected as two discrete bands, with an additional, lower molecular weight protein being visible when expression of E2 was high. The smaller bands were more evident for UKN1A14.40 (Fig. 5b), but was also present at a low level in blots of UKN1A14.38 (Fig. 5d). Detection of this band did not correlate with infectivity of particles produced from these transfections.

### Selection of pseudotype packaging vector influences infectivity of HCVpp

**Fig. 6.**
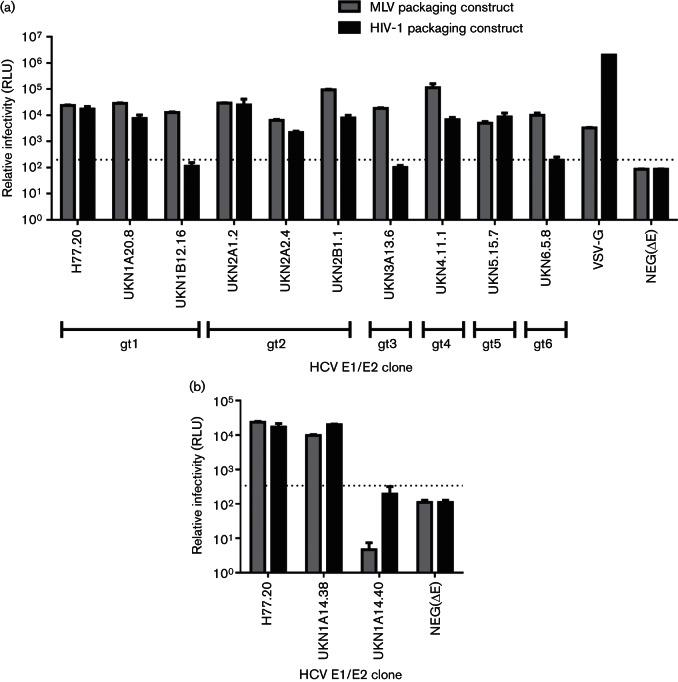
Effect of pseudoparticle backbone on HCVpp infectivity. (a) Nine patient-derived E1/E2 clones representing genotypes 1–6 which were previously selected for function using the MLV Gag/Pol packaging vector were tested using either an MLV packaging vector (grey bars) or an HIV packaging vector (black bars). A VSV-G pseudovirus preparation was performed in parallel. The threshold for a positive signal was set at three times the signal achieved with a negative (Δglycoprotein) (dotted line). Three of the nine clones were infectious using an MLV packaging vector, but non-infectious using an HIV packaging vector. (b) The two clones investigated from patient 1A14 (UKN1A14.38, UKN1A14.40) were compared with both MLV and HIV packaging vectors. UKN1A14.40 was non-functional in the context of both packaging vectors, although the signal was higher when using the HIV-1-based packaging construct. UKN1A14.38 was functional in both vectors.

Having established that the amounts of glycoprotein-encoding and MLV packaging plasmids delivered to producer cells influenced the ability to recover infectious HCVpp, the influence of the species of retrovirus used as packaging construct was investigated (Fig. 6). Using a small panel of pcDNA3.1-cloned E1/E2 sequences that have previously been determined as infectious in an MLV-based pseudotype assay (Lavillette *et al.*, 2005; Owsianka *et al.*, 2005), pseudoparticles were generated using either MLV packaging plasmids or HIV-1-based (pNL4.3) packaging plasmids. Again, large differences in infectivity were observed (Fig. 6a). Some E1/E2 sequences were equally infectious in both model systems, including H77, UKN1A20.8, UKN2A1.2 and UKN5.15.7. However, greater than 100-fold higher infectivity was observed for some clones with MLV backbones system compared to HIV-1 backbones, including UKN1B12.16, UKN3A13.6 and UKN6.5.8. While none of the HCV E1/E2 clones gave higher signal in an HIV-based backbone, infectivity of the VSV-G control pseudovirus was a >1000-fold higher signal in the HIV-1-based pseudoviruses compared to MLV-based pseudoviruses. Comparison of the infectivity of the two selected UKN1A14 clones, using the two packaging constructs, showed that the phenotype of each was comparable in the two systems (Fig. 6b). Using standard assay parameters, UKN1A14.40 did not generate a signal significantly above the assay threshold with either packaging system, while the signal achieved with UKN1A14.38 was comparable for both packaging vectors.

### Patient-derived E1/E2 glycoproteins are infectious in an HCVcc model of infection

**Fig. 7.**
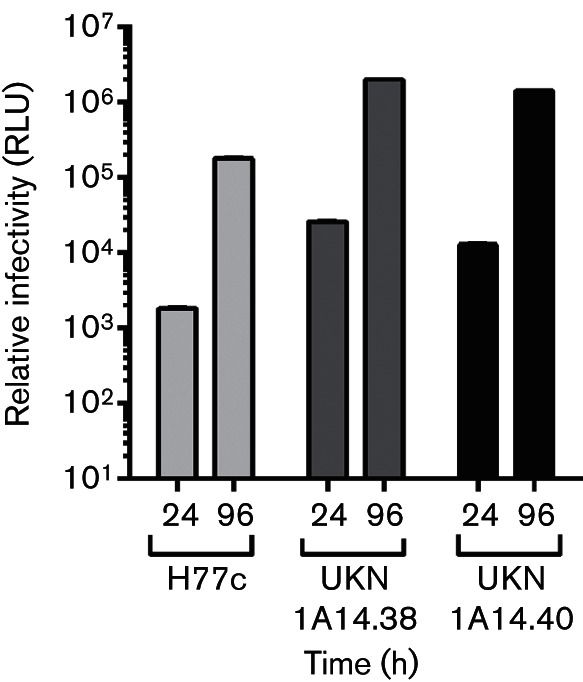
HCVcc chimeras possessing the E1/E2 glycoproteins of patient-isolated clones are infectious. Infectivity assays were performed with cell-culture supernatants of HuH7.5 cells electroporated with RNA representing H77/JFH1-luc reporter viruses possessing the E1/E2 genes of H77c (H77.20), UKN1A14.38 or UKN1A14.40. Filtered cell supernatants sampled at 24 h and 96 h were used to infect naïve Huh7.5 cells and luciferase readout measured at 24 h and 96 h after infection. Data from a mock transfected virus preparation (~800 RLU) were subtracted from each of these values.

To confirm if the E1/E2 glycoproteins facilitated infection in the context of a full-length replicating virus, HCVcc chimeras derived from an H77/JFH-1 clone (Reyes-del Valle *et al.*, 2012) were generated with the E1/E2 genes of either UKN1A14.40 or UKN1A14.38. The break points for these chimeras were at amino acid residues 171 and 734 (referenced to the H77c genome), including the polymorphisms at aa179 and aa691. Each of these clones formed infectious viruses with titres approximately 10-fold higher than the H77c control (Fig. 7). Viruses sampled 24 or 96 h after electroporation of naïve HuH7 cells had equivalent infectious titres, irrespective of the patterns of infectivity observed in the HCVpp system.

### Infectivity of pseudoviruses bearing ebolavirus glycoproteins is sensitive to changes in plasmid quantities and selection of packaging vector

**Fig. 8.**
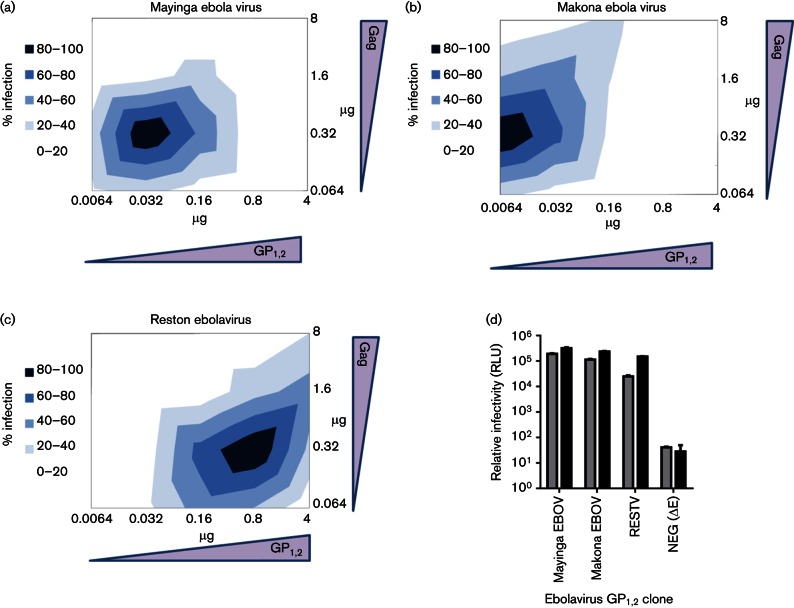
Infectivity of pseudoviruses generated with GP_1,2_ glycoproteins representing the Zaire Mayinga, Zaire Makona and Reston ebolaviruses. Transfections were performed with matrices of plasmids similar to that performed with HCV glycoproteins, using an MLV packaging construct. Infectivity was assessed by relative luminescence, and normalized to maximal signal for each glycoprotein (a, Mayinga: 30 000 RLU; b, Makona: 300 000 RLU; c, Reston: 75 000 RLU). (d) Mayinga EBOVpv, Makona EBOVpv and RESTVpv were tested using either an MLV packaging vector (grey bars) or an HIV packaging vector (black bars). While all three strains of EBOV performed similarly in the HIV-1-based vector, RESTVpv were significantly less infectious in the MLV-based pseudoviruses.

To investigate whether the patterns of infectivity observed for HCVpp generated with different amounts of plasmids also affected other virus pseudotypes, we performed the same matrix of transfections using plasmids encoding the GP_1,2_ of three ebolaviruses: Zaire ebolavirus H.sapiens-tc/COD/1976/Yambuku-Mayinga (Mayinga EBOV), Zaire ebolavirus H.sapiens-wt/GIN/2014/Makona-Gueckedou-C07 (Makona EBOV) and Reston virus M.fascicularis-tc/USA/1989/Philippines89-Pennsylvania (RESTV). Again we observed that the amount of glycoprotein construct and packaging vector plasmids used to create pseudoviruses greatly affected the ability to recover infectious particles (Fig. 8a–c). In contrast to HCVpp, the greatest infectivity of Mayinga EBOVpv occurred with much lower concentrations of the MLV packaging plasmid (0.32 µg) and the glycoprotein-encoding plasmid (0.032 µg). Makona EBOVpv required even less glycoprotein (0.0064 µg), whereas RESTV infectivity was greatest when using 0.32 µg packaging vector and 0.8 µg GP_1,2_ plasmid. We also investigated the influence of the species of retrovirus used as packaging construct (Fig. 8d). As with the HCVpp, the differences observed were strain specific, although the HIV-1 packaging construct consistently provided higher levels of infectivity. RESTVpv had a sixfold increase in infectivity with the HIV-1 backbone. Both Zaire EBOVpv strains had a twofold increase of signal with the HIV-1 backbone compared to the MLV-based pseudoviruses. These differences highlight the need to optimize transfection protocols for each individual virus before assessing relative infectivity and performing inhibition assays.

## Discussion

Retroviral pseudotypes are a flexible platform for assessing the function of glycoproteins isolated from a wide range of enveloped virus species, including hepaciviruses (Bartosch *et al.*, 2003a), filoviruses (Wool-Lewis & Bates, 1998), retroviruses (Li *et al.*, 2005), lyssaviruses (Wright *et al.*, 2009), influenza viruses (Temperton *et al.*, 2007) and arenaviruses (Vela *et al.*, 2007). However, there have been challenges to reliable recovery of infectious viruses from polymorphic HCV populations. Some studies identified high proportions of infectious patient-isolated E1/E2 clones (Dowd *et al.*, 2009; Fafi-Kremer *et al.*, 2010), while others recovered lower frequencies of virus clones that are infectious in HCVpp assay (Lavillette *et al.*, 2005; McKeating *et al.*, 2004; Urbanowicz *et al.*, 2015). As such, the use of the pseudoparticles platform might create a bias towards the selection of particular strains, which do not necessarily represent the broader range of circulating virus isolates. The data presented here offers a potential explanation for the discrepancies in the recovery frequency of infectious patient-isolated clones between studies, and develops approaches to expand the pool of functional HCVpp clones. Differences in the amounts of plasmid used when screening new patient- isolated glycoproteins might influence the functionality of the particles produced, as shown for reference clones that have previously been demonstrated to be infectious. We examined closely related E1/E2 sequences recovered from a single patient. The infectivity of the phenotype of E1/E2 clones using standard HCVpp assay parameters could be switched by a single non-synonymous nucleotide substitution in the E2 protein, highlighting the sensitivity of the system to small, biologically relevant genetic changes in populations. This is consistent with previous reports of the sensitivity of HCVpp to *in vitro* mutation (Owsianka *et al.*, 2006). Previous studies have described naturally occurring variants that were not tolerated in HCVpp entry assays (Keck *et al.*, 2009) and E1/E2 mutations that were tolerated differently in the HCVpp and HCVcc models (Goffard *et al.*, 2005; Helle *et al.*, 2010; Russell *et al.*, 2009).

In addition to the impact of the quantity of packaging construct used for HCVpp, we also observed that the species of packaging construct influences infectivity. Previous studies screening HCV glycoprotein function have generally been performed using only one of two retrovirus pseudotype platforms, utilizing either HIV-1 (Hsu *et al.*, 2003) or MLV-based backbones (Bartosch *et al.*, 2003b). Although these approaches are generally comparable in preparation, readout and interpretation, there is very little evidence of direct comparisons of these two systems with different HCV glycoproteins, although assembly has been compared in MLV and SIV-based vectors (Sandrin & Cosset, 2006). HCV glycoprotein-encoding constructs that were initially screened in the MLV-based HCVpp assay have been found to be non-infectious in the HIV-1-based HCVpp assay, leading to selection of specific clones for neutralization experiments based on their ability to give reproducible infectivity (Giang *et al.*, 2012). Methods such as ‘spinoculation’, concentration of virus preparations with sucrose cushion centrifugation or addition of polybrene have been used to improve infection of these particles (Giang *et al.*, 2012; Keck *et al.*, 2012; McKeating *et al.*, 2004). Here we demonstrated that selection of an appropriate retrovirus backbone is potentially critical to the infectivity phenotype of a patient-isolated glycoprotein construct in HCVpp. This demonstrates that infectivity phenotype cannot necessarily be compared between MLV- and HIV-based systems, and that care should be taken when investigating the effects of mutations in the two systems. However, the finding that the patient-derived clones tested here facilitated infection in the context of a chimeric HCVcc virus suggests that HCVpp systems can underestimate the infectious potential of some patient-isolated virus glycoprotein variants.

One previously described determinant of strain-dependent HCVpp infection is the presence of an intact Core protein (aa1-191) at the N-terminus of the glycoprotein construct. This has been proposed to be essential for efficient formation of pseudoparticles in a strain-dependent manner (Shukla *et al.*, 2010), with JFH-1pp requiring intact Core, but H77 requiring only the C-terminal 21 amino acids. However, it was found that infectious pseudoparticles were generated for a large number of clones, including JFH-1, when only the last 21 amino acids of Core/E1 signal peptide were included (Urbanowicz *et al.*, 2015).

The direct cause of the differences in production of infectious particles, when varying concentrations of plasmids were used to generate pseudotypes, requires further investigation. Reliable glycoprotein incorporation does not appear to be an effective predictor of HCVpp entry, as very low levels of the HCV glycoproteins (undetectable by Western blot) were able to mediate infection in our assays. This is consistent with previous analysis of sucrose-gradient sedimented HCVpp preparations, where fractions containing infectious pseudoparticles were associated with detectable HIV-1 p24, but not HCV E2 (Flint *et al.*, 2004). More sensitive assays will be required to assess the amount of glycoprotein required to mediate successful pseudotype entry. There may be many reasons for the reduction in production of infectious particles with increased glycoprotein expression. Expression could result in greater autophagy of expressed proteins, or reduced retrovirus budding. It is possible that increased expression of viral glycoproteins results in increased toxicity to the producer cells, affecting release of infectious particles. Indeed, the ebolavirus glycoproteins have been demonstrated to have toxic effects on producer cells (Sullivan *et al.*, 2005; Yang *et al.*, 2000). Greater analysis of the effects of glycoprotein expression on HEK293T cells is warranted. Mohan and colleagues have recently shown that reducing the amount of EBOV GP_1,2_ plasmid increases observed infectivity (Mohan *et al.*, 2015). Our study of HCV glycoprotein incorporation is consistent with these data and, varying the amount of the packaging vector, introduces a new factor that influences infectivity in addition to the distribution or toxicity of the glycoprotein. It is also plausible that balancing the delivery of plasmids encoding the packaging vector and glycoproteins means that there is competition for expression of the two constructs from the common promoter. This could be further investigated by using different promoters for the different elements of the pseudotype, or introducing an additional irrelevant gene under the action of a CMV promoter.

The differences in infectivity of pseudoparticles generated with HIV-1 or MLV backbones was also intriguing, suggesting that incorporation of different isolates of the HCV glycoproteins into retroviral particles is dependent on the specific assembly of the packaging virus. Subtle differences in the assembly of the two retroviruses may influence incorporation of the glycoproteins into infectious particles in an E1/E2-specific manner. For example, differences in assembly site influence incorporation of the feline endogenous retrovirus RD114 glycoprotein (Sandrin *et al.*, 2004), and the Ebola GP_1, 2_ (Leung *et al.*, 2008). HCV glycoproteins are likely to be incorporated into MLV-based pseudoparticles in an intracellular compartment. Genetic variation in the E1/E2 glycoproteins could alter their cellular localization, resulting in different interactions with cellular chaperones and subsequent incorporation into particles. Chaperone interactions are dependent on the presence of viral glycans (Choukhi *et al.*, 1998), and while the genetic variation observed in our virus population did not appear to affect the presence of N-linked glycosylation sequons, we did observe two discrete bands representing the E2 glycoprotein in our Western blots. It is plausible that these are differentially glycosylated forms of the protein, as described previously (Chapel *et al.*, 2007; Goffard *et al.*, 2005). High-resolution fluorescence microscopy would be required to resolve the fine differences in intracellular localization of these HCV glycoproteins. It will be important to perform these experiments in the context of our chimeric HCV viruses. The glycoprotein construct can influence the site of Gag/glycoprotein co-localization (Bouard *et al.*, 2007; Sandrin & Cosset, 2006), and interact with HIV-1 Vpu resulting in depleted levels of intracellular Env (Christodoulopoulos *et al.*, 2010). As a consequence, incompatibilities between some glycoprotein/packaging constructs have been observed (Christodoulopoulos & Cannon, 2001). It remains to be determined if individual viral polymorphisms affect the localization with Gag, influencing incorporation into pseudoparticles or authentic virus particles.

In summary, the demonstration that artefacts of HCVpp assays can result in underestimation of the functional capacity of patient-isolated E1/E2 glycoproteins to mediate entry into permissive cells highlights the need for better models to investigate the consequences of genetic polymorphisms on HCV entry and neutralization using panels of well-validated glycoprotein clones. We have recently described the generation of a panel of HCV E1/E2 clones that were functional in HCVpp, representing 24 % of the HCV sequences tested (Urbanowicz *et al.*, 2015). While testing different combinations of plasmid concentrations is technically laborious, future screening of new isolates and mutant E1/E2 clones for the ability to mediate entry in an HCVpp model should take this experimental artefact into account. While we have not extensively tested all of the clones we initially screened to be negative in HCVpp assay, we are now performing comparisons of HCVpp and HCVcc chimeras to determine the infectious phenotype of a much broader panel of isolates. It is likely that HCVpp when generated using optimized protocols will yield E1/E2 that is functionality more predictive of the phenotype in cell-cultured HCV. Care must also be taken when preparing pseudotypes bearing the glycoproteins of other viruses. This is particularly important in establishing entry assays for newly emerging highly pathogenic viruses that rely on pseudotypes as a model for entry.

## Methods

The E1/E2 genes were cloned from RNA extracted from the serum of chronically infected patients using previously described methods (Tarr *et al.*, 2007). PCR-amplified E1/E2 clones were ligated into the pcDNA3.1D-V5-His-TOPO vector (Life Technologies) and clones selected. Transfection-quality plasmids were prepared from transformed cultures of TOP10 cells (Life Technologies) using endotoxin-free Genelute^TM^ HP Midiprep kits (Sigma). DNA quality was assessed by agarose gel electrophoresis and quantified by spectrophotometry at 260/280 nm using a Nanodrop (Thermo). All plasmids possessed A260/A280 ratios of ~1.8.

Retroviral pseudotypes with patient-derived HCV E1/E2 clones were initially prepared using HEK293T cells as described previously (Tarr *et al.*, 2007), based on the protocols described by Bartosch and colleagues (Bartosch *et al.*, 2003a, b). A luciferase-encoding reporter plasmid (pTG126) and an MLV Gag/Pol-encoding packaging construct (phCMV-5349) were kind gifts from François-Loïc Cosset. Briefly, 1.2×10^6^ HEK293T cells were seeded overnight in a 10 cm diameter Primaria-coated dish (Corning) in 10 ml of Dulbecco's Modified Eagle Medium (DMEM, Gibco) supplemented with non-essential amino acids and heat-inactivated FBS (both Gibco). Transfections were performed with 2 µg of each of three plasmids (Fig. 1) using 24 µl cationic polymer transfection reagent (Polyethylenimine), in the presence of Optimem (Gibco), and the media replaced with 10 ml complete DMEM after 6 h. Pseudoparticle-containing supernatants were recovered after 72 h and passed through a 0.45 µM filter. From this, 100 µl aliquots were used to infect 1.5×10^3^ HuH7 cells for 4 h in a 96-well white plate (Corning). Following infection 200 µl DMEM was added to the cells. Seventy-two hours following infection, media was discarded, cells lysed with 50 µl Cell Lysis Buffer (Promega) and luminescence assessed for each infection using a BMG Labtech FluoroStar Omega luminometer. PMT gain was set to 3600 and 50 µl of Promega luciferase substrate was injected immediately before a 1 s luminescence reading. Using this protocol, negative control readings following addition of pseudotypes bearing no glycoproteins routinely resulted in a luminescence between 0 and 100 relative light units (RLUs), dependent on the experiment. A total of 493 different E1/E2-encoding plasmids isolated from 63 infected patients were screened for infectivity in HuH7 cells. Assigning a threshold of three times the background signal achieved in the presence of pseudoparticles generated without glycoproteins, 118 were defined as infectious (Fig. 2). Subsequent analysis was performed with clones representing viruses H77 (GenBank accession AF011751), UKN2A1.2 (AY734977), UKN1A14.40 (AY958061) and UKN1A14.38 (AY958060). Particles were then produced using varying amounts of both the E1/E2-encoding plasmid and the MLV packaging plasmid to optimize expression of each of the component proteins, with a fixed amount (2 µg ml^−1^) of the reporter plasmid. Plasmid quantities were between 8 and 0.0064 µg. In some experiments, a pNL4.3 HIV-1 retrovirus backbone was used to manufacture pseudoparticles as previously described (Hsu *et al.*, 2003; McKeating *et al.*, 2004). Dose-dependent HCVpp neutralization experiments were performed with the cross-neutralizing monoclonal antibody AP33 as previously described (Owsianka *et al.*, 2005). Infectivity plots were normalized to the greatest luciferase signal achieved for each glycoprotein construct (100 %), and the luciferase signal achieved with a control pseudoparticle preparation with glycoprotein plasmid omitted (0 %) using Graphpad Prism 6.0. These data were presented using Microsoft Excel 2010 as three-dimensional plots with E1/E2-encoding plasmid concentration on the *x*-axis, normalized luciferase signal on the *y*-axis and packaging vector plasmid concentration on the *z*-axis.

Pseudoviruses, using similar experimental conditions to the HCVpp, were also generated with glycoproteins corresponding to Zaire ebolavirus H.sapiens-tc/COD/1976/Yambuku-Mayinga (Mayinga EBOV; GenBank accession NC_002549) and Reston ebolavirus M.fascicularis-tc/USA/1989/Philippines89-Pennsylvania (RESTV; GenBank accession NC_004161), (Kobinger *et al.*, 2001) both were kind gifts from Gary Kobinger. A codon optimized construct for Zaire ebolavirus H.sapiens-wt/GIN/2014/Makona-Gueckedou-C07 (Makona EBOV; GenBank accession KJ660347.2) was also used, a kind gift from Etienne Simon-Loriere. The ebolavirus glycoprotein-encoding genes were cloned into the pcDNA3.1 plasmid and co-transfected with pTG126 and phCMV-5349. Ebolavirus pseudoviruses (EBOVpv) were used to infect HuH7 cells similarly to HCVpp.

HCV pseudoparticles were pelleted by ultracentrifuging cell supernatants through a 20 % sucrose cushion at 160  000 *g* (40 000 rpm in a Beckman Type 70 Ti rotor) for 150 min at 4°C. Pellets were resuspended in 50 µl PBS for analysis by Western blotting. Antibodies AP33 and ALP98 (Owsianka *et al.*, 2001) (kind gifts from Arvind Patel) and a polyclonal rabbit anti-MLV capsid (a kind gift from Jean Dubuission) were used to detect pseudoparticle-associated proteins after separation on a 10 % polyacrylamide gel.

HCVcc E1/E2 chimeras were generated as previously described (McClure *et al.*, 2016) in a genotype 1a Bi-gluc-H77c (1a)/JFH(T2700c A4080T) chimeric virus (Reyes-del Valle *et al.*, 2012) (a kind gift of Charles Rice), encoding the Core/p7/NS2 of strain H77 and the E1/E2 glycoproteins of viruses screened by HCVpp assay. These constructs possessed a Gaussia luciferase reporter. Infection assays with chimeric viruses were performed essentially as previously described (Lindenbach *et al.*, 2005), determining infection by measuring luciferase expression with a Biolux Gaussia Lucierase assay kit (NEB).
